# Adaptive Laboratory Evolution of the Non-Conventional Yeast *Pichia ethanolica* for Improved Molasses Utilization Reveals Multilevel Adaptive Remodeling

**DOI:** 10.3390/microorganisms14092037

**Published:** 2026-09-12

**Authors:** Peng Yang, Yutian Fan, Bingqi Hu, Wencan Ren, Chenlu Shi, Yufei Jiang, Borong Li, Xinyue Zhang, Hao Liu, Zhen Chen, Andong Gong

**Affiliations:** 1 Dabie Mountain Laboratory, College of Tea and Food Science, Xinyang Normal University, Xinyang 464000, China; yangpeng@xynu.edu.cn (P.Y.); fanyutian2001@163.com (Y.F.); 17538695956@139.com (B.H.); lbr200695@outlook.com (B.L.); 15535312052@163.com (X.Z.); haoliu@xynu.edu.cn (H.L.); chenzhen@xynu.edu.cn (Z.C.); 2 Henan Key Laboratory of Tea Plant Biology, College of Tea and Food Science, Xinyang Normal University, Xinyang 464000, China; 3 Henan International Joint Laboratory of Tea-Oil tree Biology and High-Value Utilization, College of Tea and Food Science, Xinyang Normal University, Xinyang 464000, China; 4 College of Life Science, Xinyang Normal University, Xinyang 464000, China; 17839186593@163.com (W.R.); 18568841203@163.com (C.S.); 18450026192@163.com (Y.J.)

**Keywords:** *Pichia ethanolica*, adaptive laboratory evolution, molasses utilization, stress tolerance, multi-omics

## Abstract

Molasses is an abundant and low-cost fermentation feedstock, but its direct utilization is often constrained by its complex composition and inhibitory components. In this study, a molasses-utilizing yeast strain, M0, was isolated from tea rhizosphere soil and identified as *Pichia ethanolica*. M0 grew directly in untreated molasses diluted only with water, and adaptive laboratory evolution (ALE) was performed under stepwise increasing molasses concentrations. After 70 transfers, the evolved population reached an OD_560_ of 26.41, representing a 73.1% increase relative to the parental strain, and a superior isolate was designated M70. M70 showed lower residual sucrose after 48 h in 100 g/L molasses medium (16.02 vs. 21.11 g/L) and improved growth under high-molasses, -temperature, and -NaCl conditions, with additional growth advantages under selected ethanol and oxidative-stress conditions. Genome resequencing identified five small candidate variants and one interchromosomal duplicative insertion, including alterations involving *RIB1*, *SOL3*, and a *CET1*-like/*REX2*-like region. Transcriptomic and metabolomic analyses identified 179 differentially expressed genes and 169 differentially accumulated metabolites. Integrated analysis highlighted coordinated changes in D-amino acid, arginine and proline, beta-alanine, and glyoxylate and dicarboxylate metabolism. These findings indicate multilevel genomic, transcriptional, and metabolic remodeling associated with improved molasses adaptation in *P. ethanolica*.

## 1. Introduction

Molasses, a by-product of sugar refining, has been widely used as an economical substrate for microbial fermentation because of its high sucrose content and abundant nutrients [1]. However, crude molasses contains various inhibitory components, such as heavy metals, pigments, and suspended solids, which can suppress microbial growth and reduce product formation, thereby increasing pretreatment costs and limiting overall process efficiency [2]. Beyond these inhibitory effects, molasses utilization is also constrained by the capacity of microorganisms to efficiently assimilate its carbon sources and adapt to the complex, high-sugar environment. Therefore, improving microbial utilization of molasses remains an important challenge in industrial fermentation.

To improve molasses utilization efficiency, metabolic engineering strategies have been extensively explored to optimize carbon metabolism, rebalance metabolic flux, alleviate carbon catabolite repression, and enhance cellular stress tolerance. These strategies have targeted multiple regulatory and metabolic processes, including stress-responsive pathways such as the HOG pathway [3], global carbon regulation and sugar transport involving Cra and PtsG [4], and the reconstruction of sucrose-utilization modules such as the *csc* operon [5]. In addition, heterologous expression of sucrose-utilization genes, including the sucrose transporter gene *StSUT1* and the invertase gene *SUC2* [6], as well as the introduction of sucrose-specific pathways involving *scrA* and *scrB* together with global regulatory modifications of *rex*, *argR*, and *fur* [7,8], has further expanded the capacity of diverse microbial hosts to utilize sucrose-rich substrates.

Despite these advances, rational engineering strategies remain constrained by their reliance on detailed genetic information and their limited ability to account for the complexity of cellular regulatory networks [9]. Moreover, efficient molasses fermentation requires microorganisms to maintain effective carbon-source utilization while simultaneously coping with inhibitors, osmotic pressure, and other environmental stresses. Interactions among these factors can generate physiological trade-offs that are difficult to address through targeted engineering alone. Therefore, complementary non-targeted approaches capable of improving complex phenotypes at the whole-cell level are needed, among which adaptive laboratory evolution (ALE) has emerged as a promising strategy.

Through continuous cultivation under defined selective pressures, ALE promotes the gradual accumulation of beneficial mutations and enables whole-cell adaptation to environmental and metabolic constraints [10]. In terms of substrate utilization and fermentation performance, ALE has been applied to improve sugar utilization and product formation in molasses-based fermentations, including DHA production by *Schizochytrium* sp. and ethanol production by *Zymomonas mobilis* [11,12]. ALE has also been used to enhance microbial tolerance to stresses relevant to molasses fermentation, including osmotic stress, ethanol stress, high temperature, and the combined inhibitory effects of elevated K^+^ and Ca^2+^ concentrations [13]. These adaptive phenotypes are often accompanied by extensive metabolic and regulatory remodeling involving central carbon metabolism, cofactor supply, transport processes, and stress-response pathways across diverse microbial hosts [14,15]. Nevertheless, how ALE reshapes metabolic and regulatory networks to improve molasses utilization, particularly in non-conventional yeasts, remains insufficiently understood.

Accordingly, in this study, a yeast strain M0 was isolated from soil and identified as *Pichia ethanolica*. ALE was subsequently applied to improve its molasses utilization capacity and stress tolerance, resulting in the evolved strain M70 with enhanced growth performance under high-molasses conditions. Whole-genome sequencing of M0 was performed for genomic characterization and to provide a reference for subsequent analyses, while comparative whole-genome resequencing and transcriptomic and metabolomic analyses were conducted to characterize the genetic, transcriptional, and metabolic changes associated with adaptive evolution. This study provides insights into the molecular basis of ALE-mediated adaptation in a non-conventional yeast and highlights the potential of adaptive evolution for improving microbial performance in molasses-based fermentation.

## 2. Materials and Methods

### 2.1. Strains and Media

*P*. *ethanolica* M0, isolated and identified in this study, was used as the parental strain for adaptive laboratory evolution. The evolved strain M70 was obtained after long-term adaptive laboratory evolution under progressively increasing molasses concentrations. The media used for strain cultivation and phenotypic characterization are described below.

YPD medium was used for seed culture and contained, per liter, 10 g yeast extract, 20 g peptone, and 20 g glucose.

Molasses medium was prepared by directly diluting sugarcane molasses (Solarbio, Beijing, China) with distilled water to the desired concentrations without additional pretreatment. The molasses contained 8.3% (*w*/*w*) glucose, 28.6% (*w*/*w*) sucrose, 12.1% (*w*/*w*) fructose, 2.1% (*w*/*w*) other carbohydrates, 2.9% (*w*/*w*) crude protein, 9.05% (*w*/*w*) ash, and 5.3% (*w*/*w*) metal ions, with a pH of 5.90.

To compare the growth performance of M0 and M70 on different individual carbon sources, synthetic media containing glucose, fructose, or sucrose as the sole carbon source were prepared. Each liter of synthetic medium contained 40 g of the respective carbon source, 7.8 g (NH_4_)_2_SO_4_, 0.53 g MgSO_4_·7H_2_O, and 1.5 g KH_2_PO_4_.

Solid media were prepared by adding 2% (*w*/*v*) agar to the corresponding liquid media. All media were sterilized at 121 °C for 20 min before use.

### 2.2. Isolation of Strain M0

Soil samples were collected from the tea rhizosphere in Xinyang, Henan Province, China, at a depth of approximately 10 cm. For enrichment, 1 g of soil was suspended in 9 mL of sterile water, and the suspension was inoculated at 1% (*v*/*v*) into 50 mL of 4% (*w*/*v*) molasses medium in 250 mL Erlenmeyer flasks. Cultures were incubated at 30 °C and 150 rpm for 48 h and transferred three consecutive times into fresh molasses medium at a 1% (*v*/*v*) inoculum. The enriched cultures were serially diluted and spread onto 4% (*w*/*v*) molasses agar plates for colony isolation.

Representative colonies with distinct morphologies were selected, and 20 isolates were screened in liquid molasses medium. Each isolate was cultivated in 5 mL of 4% (*w*/*v*) molasses medium at 30 °C and 150 rpm for 48 h, after which OD_560_ was measured. The isolate with the highest OD_560_ was designated as strain M0 and used for subsequent experiments.

### 2.3. Carbon Source Utilization Profiling of Strain M0

Carbon source utilization of strain M0 was characterized using Biolog YT MicroPlates (Biolog, Hayward, CA, USA) according to the manufacturer’s instructions. Briefly, strain M0 was cultured on BUY (Biolog Universal Yeast) agar at 26 °C, and fresh colonies were suspended in sterile water to approximately 47% transmittance. The standardized cell suspension was inoculated into YT MicroPlates (catalog no. 1005) at 100 μL per well. The plates were incubated at 26 °C for 24–72 h until metabolic profiles developed. Plate responses at 590 nm were recorded using the Biolog MicroLog System to generate the carbon source utilization profile of strain M0.

### 2.4. Molecular Identification and Genome Sequencing of Strain M0

A fragment spanning the partial 18S rRNA gene and internal transcribed spacer (ITS) region was amplified by PCR using primers NS3 (5′-GCAAGTCTGGTGCCAGCAGCC-3′) and ITS4 (5′-TCCTCCGCTTATTGATATGC-3′). The resulting sequence was compared with sequences deposited in the GenBank database using BLAST (version 2.17.0). Closely related sequences were selected for phylogenetic analysis, and a phylogenetic tree was constructed using the neighbor-joining method with 1000 bootstrap replicates in MEGA version 11 [16].

To further support taxonomic identification at the genomic level, whole-genome sequencing of strain M0 was performed by Wuhan Benagen Technology Co., Ltd. (Wuhan, China). The resulting genome assembly was used as the reference genome for subsequent comparative and multi-omics analyses. Average nucleotide identity (ANI) values between strain M0 and the reference genome of *P. ethanolica* M2 (GenBank assembly accession no. GCA_001649435.1) were calculated using FastANI and skani [17,18].

### 2.5. Adaptive Laboratory Evolution

Molasses was used as the sole nutrient source for adaptive laboratory evolution. Prior to ALE, the growth of strain M0 was evaluated in molasses media containing 40–200 g/L molasses over 72 h. Based on these preliminary growth profiles, 80 g/L was selected as the initial concentration for ALE to ensure robust growth while allowing subsequent stepwise increases in selection pressure. The molasses concentration was increased by 40 g/L every 15 transfers until reaching 200 g/L, after which it was maintained at 200 g/L for the remainder of the experiment. Cultures were incubated at 30 °C and 150 rpm and serially transferred into fresh molasses medium every 72 h at an inoculum size of 1% (*v*/*v*) for a total of 70 transfers. The evolution experiment was performed with two independent lineages. Cell growth was monitored by measuring OD_560_ at the end of each transfer.

At 15-transfer intervals, cultures were serially diluted and plated for single-colony isolation, and representative isolates were preserved as glycerol stocks. After 70 transfers, 20 single-colony isolates were obtained and screened for growth in molasses medium. The isolate exhibiting the highest OD_560_ was designated as strain M70 and used for subsequent experiments.

### 2.6. Phenotypic Characterization of Strains M0 and M70

The parental strain M0 and evolved strain M70 were compared under different stress conditions to evaluate their phenotypic tolerance. Unless otherwise specified, stress assays were performed in 100 g/L molasses medium at 30 °C and 150 rpm for 48 h. The tested conditions included molasses concentrations of 100, 200, and 280 g/L, temperatures of 28, 33, and 37 °C, H_2_O_2_ concentrations of 1, 2, and 3 mM, ethanol concentrations of 5%, 10%, and 15% (*v*/*v*), and NaCl concentrations of 1%, 3%, and 5% (*w*/*v*). For the temperature assays, cultures were incubated at the indicated temperatures. Growth was evaluated by measuring OD_560_ after 48 h.

To compare growth on individual carbon sources, M0 and M70 were cultivated in synthetic media containing glucose, fructose, or sucrose at 40 g/L as the sole carbon source. Cultures were incubated at 30 °C and 150 rpm for 48 h, and growth was evaluated by measuring OD_560_.

### 2.7. Whole-Genome Resequencing and Variant Identification

Whole-genome resequencing of strains M0 and M70 was performed on the Illumina platform, with two resequencing datasets generated for M70. Raw reads were filtered using fastp (v1.0.1) and aligned to the assembled M0 reference genome using bwa-mem2 (v2.2.1). SNPs were identified using GATK (v4.6.0.0) and filtered and annotated using vcftools (v0.1.17) and ANNOVAR (20200607). Structural variations were detected using smoove (v0.2.6), delly (v1.2.6), and manta (v1.6.0), and copy number variations were analyzed using CNVpytor (v1.3.1).

Genome heterozygosity and ploidy of M0 were assessed from 21-mer distributions using GenomeScope2.0. Candidate variants associated with adaptive evolution were identified by excluding variants present in M0 and prioritizing those consistently detected in the two M70 resequencing datasets, with predicted functional effects and transcriptomic changes considered in subsequent interpretation.

### 2.8. Transcriptome Sequencing and Analysis

Strains M0 and M70 were cultivated in 100 g/L molasses medium at 30 °C and 150 rpm for 24 h, with three biological replicates per strain. Cells were harvested by centrifugation for RNA extraction and transcriptome sequencing.

Raw reads were filtered using fastp (v0.23.2) and assessed using FastQC (v0.11.9). Clean reads were mapped to the M0 reference genome using STAR (v2.7.10a), and gene expression was quantified using RSEM (v1.3.1). Differentially expressed genes (DEGs) were identified from read counts using DESeq2 (v1.34.0), with |log_2_ (fold change)| > 1 and FDR < 0.05. GO and KEGG enrichment analyses of DEGs were performed using clusterProfiler (v4.2.2).

### 2.9. Metabolomic Analysis

Untargeted intracellular metabolomic profiling was performed for strains M0 and M70 cultured in 100 g/L molasses medium at 30 °C and 150 rpm for 24 h, with three biological replicates per strain. Cell pellets were extracted with pre-chilled methanol/acetonitrile/water (2:2:1, *v*/*v*/*v*) containing internal standards. After homogenization, sonication, and centrifugation, the supernatants were subjected to LC-MS/MS analysis.

Metabolomic analysis was performed using a Vanquish UHPLC system coupled to an Orbitrap Exploris 120 mass spectrometer (Thermo Fisher Scientific, Waltham, MA, USA) equipped with a Waters ACQUITY UPLC BEH Amide column (2.1 × 50 mm, 1.7 μm). Raw data were processed using an XCMS-based workflow, and metabolites were annotated against an in-house metabolite database (v4.0). Principal component analysis (PCA) was performed to evaluate overall metabolic differences between M0 and M70. OPLS-DA was used to calculate variable importance in projection (VIP) scores. Differential metabolites were identified based on VIP > 1 and Student’s *t*-test (*p* < 0.05), followed by KEGG pathway enrichment analysis.

### 2.10. Analytical Methods

To minimize interference from the color of molasses, cells were harvested by centrifugation at 13,000× *g* for 5 min and resuspended in sterile water to the original culture volume. OD_560_ was then measured using a UV-1800 spectrophotometer (Shimadzu, Kyoto, Japan).

Culture samples were centrifuged, and the supernatants were filtered through 0.22 μm membrane filters prior to HPLC analysis. Organic acids, glycerol, and ethanol were quantified using an LC-20A HPLC system equipped with an RID-10A refractive index detector and an Aminex HPX-87H column (Bio-Rad, Hercules, CA, USA). The mobile phase was 5 mM H_2_SO_4_ at a flow rate of 0.6 mL/min. The column temperature was 65 °C, and the injection volume was 10 μL.

Sucrose, glucose, and fructose were analyzed using the same HPLC system equipped with a ZORBAX NH_2_ column (4.6 × 250 mm, 5 μm; Agilent Technologies, Santa Clara, CA, USA), with acetonitrile–water (75:25, *v*/*v*) as the mobile phase at 1.0 mL/min. The column temperature was 35 °C, and the injection volume was 5 μL.

### 2.11. Statistical Analysis

Data are presented as the mean ± standard deviation (SD) from at least three biological replicates. Statistical analyses were performed using SPSS version 13.0 (SPSS Inc., Chicago, IL, USA). Where applicable, the effects of strain and treatment were analyzed by two-way analysis of variance (ANOVA), followed by Tukey’s multiple-comparison test. Differences were considered statistically significant at *p* < 0.05.

## 3. Results

### 3.1. Isolation, Identification, and Characterization of the Molasses-Utilizing Yeast Strain M0

To isolate microorganisms capable of growing on molasses, tea-rhizosphere soil samples were enriched using untreated molasses as the sole nutrient source and subsequently plated on molasses agar for colony isolation. A total of 268 single colonies were obtained, from which 20 isolates with distinct colony morphologies and relatively robust growth were selected for further screening in liquid molasses medium (Figure S1). Among them, strain M0 exhibited the highest OD_560_ after 48 h of cultivation and was therefore selected for subsequent characterization and adaptive laboratory evolution.

**Figure 1:**
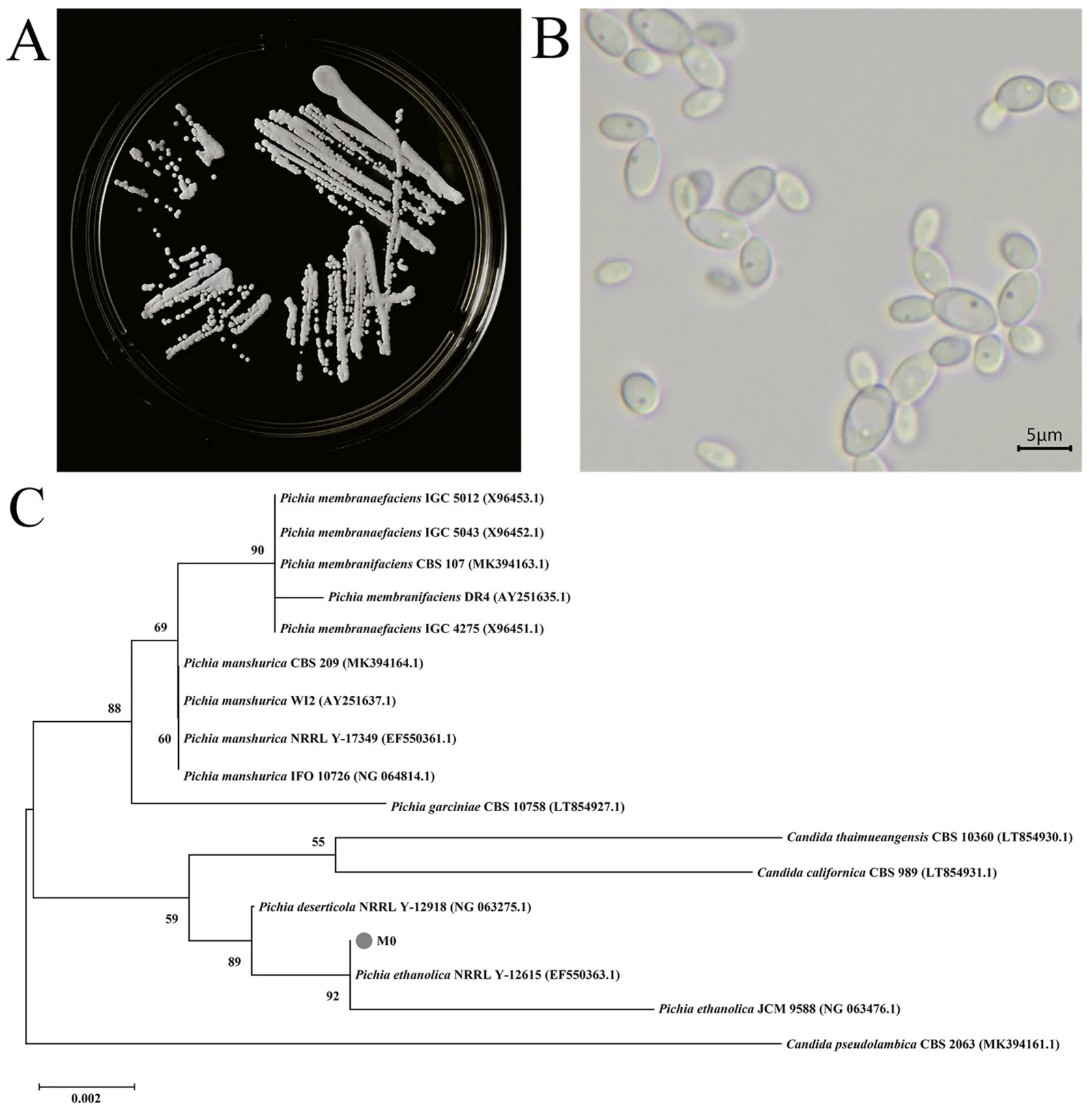
Morphological and molecular identification of strain M0. (**A**) Colony morphology of strain M0 on YPD agar; (**B**) microscopic morphology of strain M0 cells; (**C**) phylogenetic tree of strain M0 and closely related yeast strains based on the ribosomal DNA fragment spanning the partial 18S rRNA gene and ITS region. Bootstrap values (%) based on 1000 replicates are indicated at the nodes.

On YPD agar, strain M0 formed white, circular colonies with smooth, moist surfaces and regular margins (Figure 1A). Microscopic observation showed oval-shaped cells with typical budding morphology (Figure 1B), consistent with the general morphological characteristics of yeast.

For taxonomic identification, phylogenetic analysis was performed using the ribosomal DNA fragment spanning the partial 18S rRNA gene and ITS region. Strain M0 clustered closely with *Pichia ethanolica* reference sequences EF550363.1 and NG063476.1 (Figure 1C). Genome-level identification further supported this assignment, with ANI values of 98.9% and 99.2% relative to *P. ethanolica* M2 as determined by FastANI and skani, respectively. Based on the morphological, phylogenetic, and genome-level evidence, strain M0 was identified as *P. ethanolica*.

Carbon source utilization profiling using Biolog YT MicroPlates showed that strain M0 was able to utilize a broad range of substrates, including sugars, sugar alcohols, and organic acids (Table 1). Among the tested substrates, α-D-glucose exhibited the highest relative utilization level, whereas sucrose and several other carbohydrates showed moderate utilization. Strain M0 also utilized glycerol and several organic acids, indicating a relatively broad carbon utilization profile. The ability to utilize glucose and sucrose, two major fermentable sugars in sugarcane molasses, further supported the suitability of strain M0 for subsequent molasses-based cultivation and adaptive laboratory evolution.

### 3.2. Adaptive Laboratory Evolution and Selection of a Molasses-Adapted Strain M70

**Figure 2:**
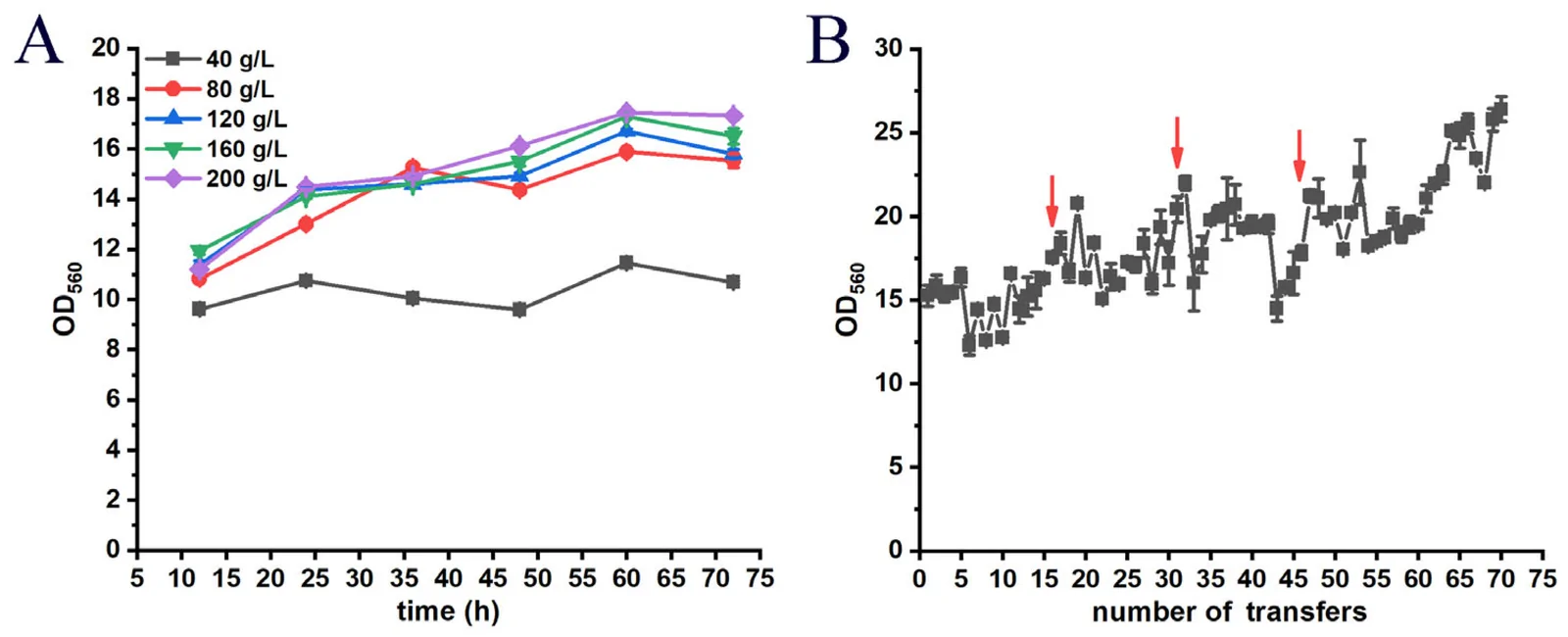
Growth of strain M0 at different molasses concentrations and changes in cell growth during adaptive laboratory evolution. (**A**) Growth curves of strain M0 in molasses media containing different molasses concentrations; (**B**) changes in OD_560_ during adaptive laboratory evolution. Red arrows indicate stepwise increases in molasses concentration by 40 g/L every 15 transfers.

To establish an appropriate starting condition for ALE, the growth of strain M0 was first evaluated at molasses concentrations ranging from 40 to 200 g/L (Figure 2A). Strain M0 maintained robust growth over a broad range of molasses concentrations, with no marked reduction in growth between 80 and 200 g/L. Based on these growth profiles, 80 g/L was selected as the initial molasses concentration for ALE.

During ALE, cultures were serially transferred every 72 h, and the molasses concentration was increased by 40 g/L every 15 transfers until reaching 200 g/L, after which it was maintained at 200 g/L until the completion of 70 transfers. Overall, growth performance progressively improved during the evolution process (Figure 2B). Stepwise increases in molasses concentration were accompanied by transient fluctuations in OD_560_, followed by gradual recovery and stabilization, indicating adaptation to the imposed selection conditions. After 70 transfers, the evolved population reached a final OD_560_ of 26.41, representing a 73.1% increase compared with the parental strain M0.

To obtain a superior evolved isolate, the final evolved population was subjected to single-colony isolation, and 20 relatively large colonies were further screened in liquid molasses medium. Among them, isolate No. 4 exhibited the highest OD_560_ after 48 h of cultivation and was designated as strain M70 for subsequent analyses (Figure S2A). M70 also showed improved growth compared with M0 in YPD medium (Figure S2B), indicating that its enhanced growth performance was not restricted to molasses-based conditions. The improved growth phenotype of M70 remained stable over ten additional serial transfers, with no significant decline in final OD_560_ values observed during the tested period (Figure S3).

**Figure 3:**
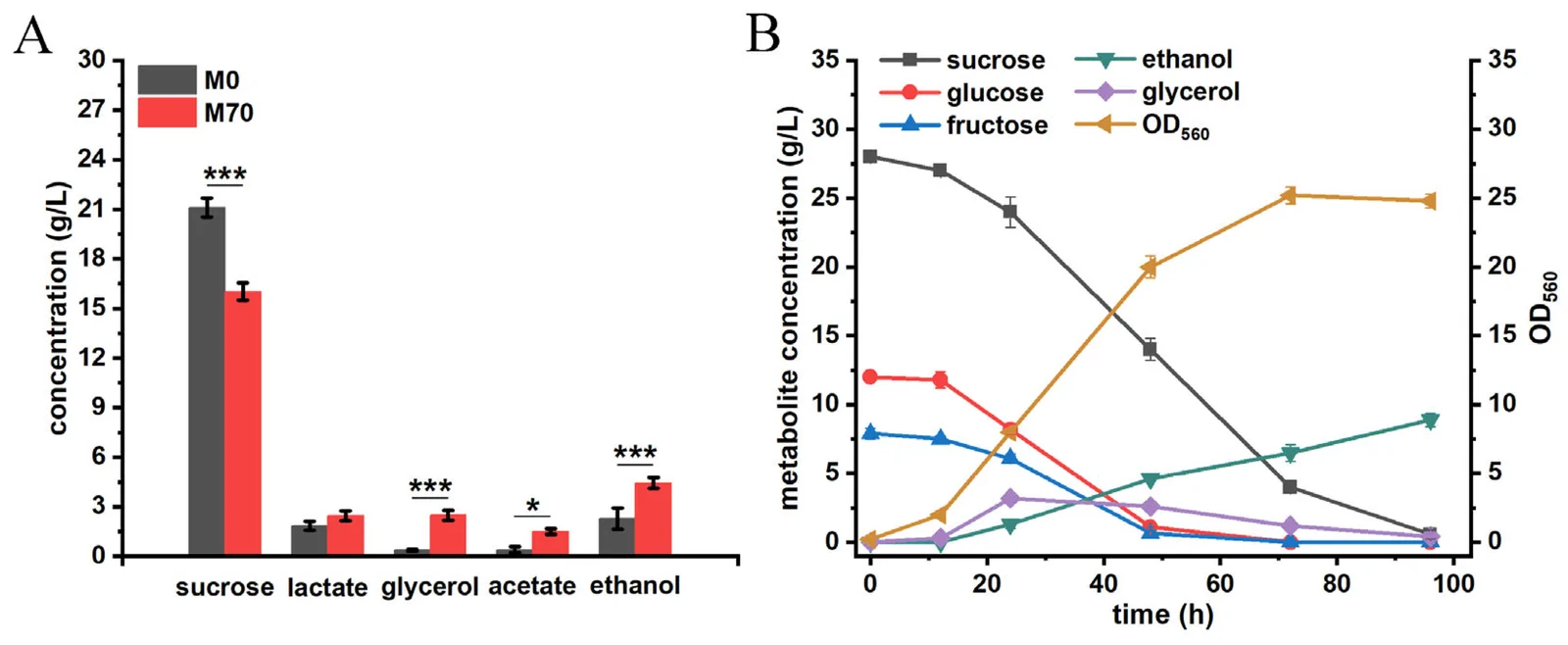
Extracellular metabolite profiles of strain M70 during molasses cultivation and comparison with the parental strain M0. (**A**) Comparison of residual sucrose and major extracellular metabolites between strains M0 and M70 after 48 h of cultivation in 100 g/L molasses medium; (**B**) time-course changes in OD_560_ and extracellular metabolites of strain M70 during 96 h of cultivation in 100 g/L molasses medium. Data are presented as mean ± standard deviation. Asterisks indicate significant differences between M0 and M70 for the same metabolite (* *p* < 0.05, *** *p* < 0.001).

To further characterize the fermentation differences between M0 and M70 under molasses cultivation, residual sugars and major extracellular metabolites were analyzed by HPLC after 48 h of cultivation in 100 g/L molasses medium (Figure 3A). Glucose and fructose were depleted to undetectable levels in both strains, whereas M70 showed a lower residual sucrose concentration than M0 (16.02 vs. 21.11 g/L). M70 also accumulated higher concentrations of glycerol (2.48 vs. 0.38 g/L) and ethanol (4.44 vs. 2.27 g/L). These results indicate that ALE altered sugar consumption and extracellular metabolite profiles during molasses cultivation.

The fermentation dynamics of M70 were further monitored over 96 h in 100 g/L molasses medium (Figure 3B). Sucrose, glucose, and fructose were progressively consumed and were nearly depleted by 96 h, demonstrating the ability of M70 to utilize the major fermentable sugars present in molasses. Cell growth increased rapidly during the first 48 h, reached a maximum OD_560_ of 25.2 at 72 h, and subsequently stabilized. Ethanol concentration increased throughout cultivation, reaching 8.9 g/L at 96 h. In contrast, glycerol accumulated transiently, reaching a maximum concentration of 3.2 g/L at 24 h before decreasing to 0.4 g/L by 96 h.

### 3.3. Enhanced Stress Tolerance and Growth Performance of Strain M70

**Figure 4:**
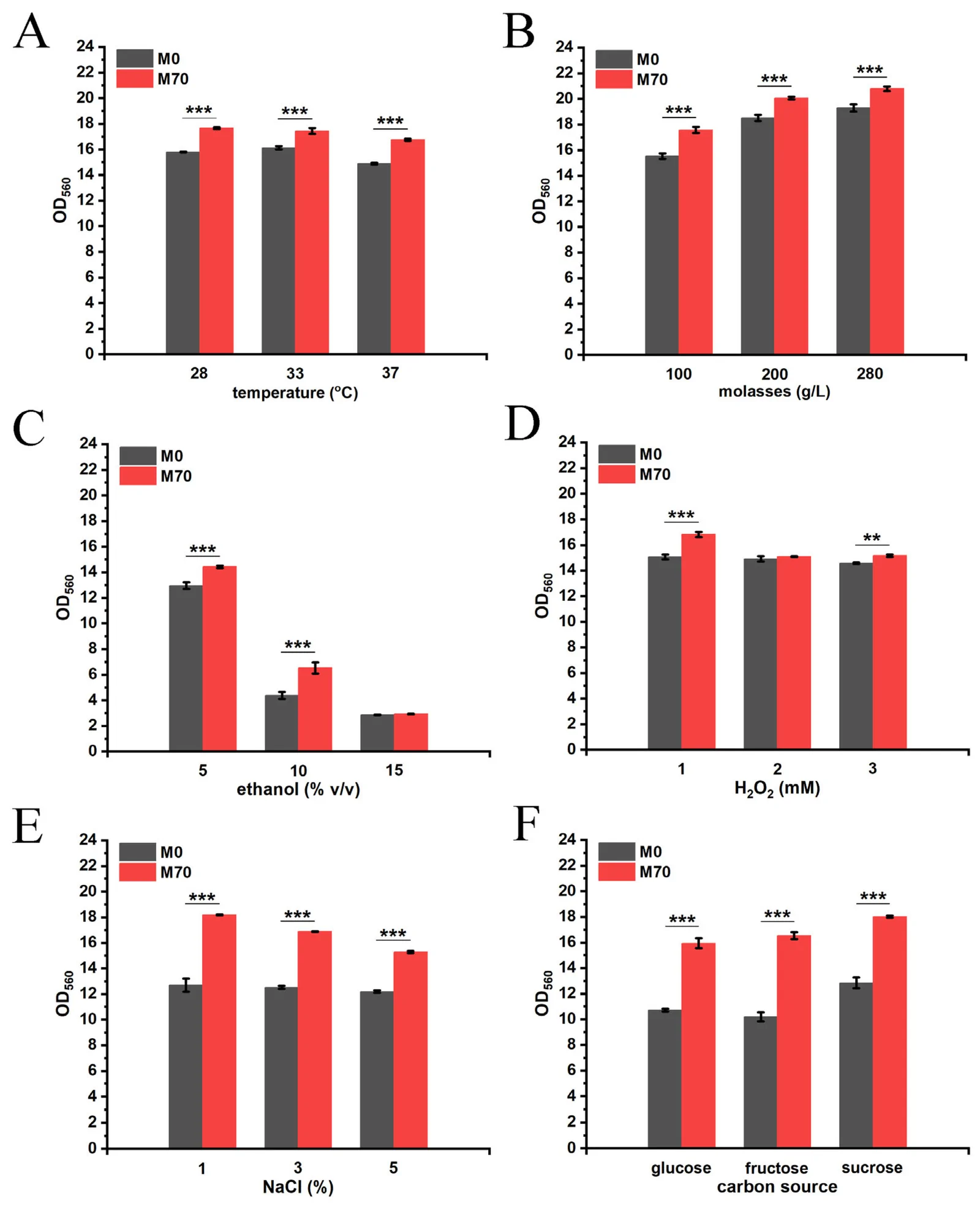
Comparative growth performance of strains M0 and M70 under different cultivation conditions. (**A**–**E**) Growth performance of strains M0 and M70 under different stress conditions: temperature (**A**), molasses concentration (**B**), ethanol (**C**), H_2_O_2_-induced oxidative stress (**D**), and NaCl stress (**E**). Except for the molasses-concentration assay, stress experiments were conducted in medium containing 100 g/L molasses. (**F**) Growth performance of strains M0 and M70 in synthetic media containing 40 g/L glucose, fructose, or sucrose as the sole carbon source. Data are presented as mean ± standard deviation. Asterisks indicate significant differences between M0 and M70 under the same condition (** *p* < 0.01, *** *p* < 0.001).

Molasses-based fermentation can expose yeast cells to multiple environmental stresses, including high osmotic pressure caused by elevated sugar concentrations, ethanol accumulation, oxidative stress, temperature variation, and ionic stress associated with molasses components. Therefore, the growth performance of M0 and M70 was compared under these representative stress conditions to evaluate phenotypic changes associated with ALE (Figure 4A–E). Overall, M70 exhibited improved growth performance compared with M0 under most tested conditions.

M70 showed significantly higher OD_560_ values than M0 at all tested temperatures (28–37 °C) (Figure 4A) and molasses concentrations (100–280 g/L) (Figure 4B), indicating improved growth across a broad temperature range and under high-molasses conditions. Ethanol supplementation inhibited the growth of both strains, whereas M70 maintained significantly higher OD_560_ values than M0 at 5% and 10% ethanol; no significant difference was observed at 15% ethanol (Figure 4C). Under oxidative stress, M70 showed significantly higher OD_560_ values than M0 at 1 and 3 mM H_2_O_2_, while no significant difference was observed at 2 mM H_2_O_2_ (Figure 4D). Under NaCl stress, M70 consistently exhibited higher OD_560_ values than M0 at all tested concentrations (1–5%) (Figure 4E), indicating enhanced salt tolerance.

In addition to stress tolerance, the growth performance of M0 and M70 was compared in synthetic media containing glucose, fructose, or sucrose as the sole carbon source. M70 exhibited significantly higher OD_560_ values than M0 under all three conditions (Figure 4F), indicating improved growth on the major sugar components of molasses. Collectively, these results show that ALE enhanced the stress tolerance and growth performance of strain M70 across diverse cultivation conditions.

### 3.4. Whole-Genome Resequencing Revealed Candidate Variants Associated with Adaptive Evolution

To identify genetic changes associated with the evolved phenotype of M70, whole-genome resequencing was performed using the M0 genome as the reference. Genome composition analysis indicated that M0 had a highly heterozygous, diploid-like genome structure (Figure S4).

Considering the heterozygous genomic background of M0, candidate variants were prioritized based on allele support, consistency between the two M70 resequencing datasets, predicted functional effects, and comparison with the parental background. Five small variants and one high-confidence interchromosomal duplicative insertion were retained as candidate variants associated with adaptive evolution (Table 2).

The small variants included an intronic mutation in a *FLO11*-like gene and nonsynonymous substitutions in *RIB1* (GTP cyclohydrolase II), *SOL3* (6-phosphogluconolactonase), an ML-like protein gene, and an ABC transporter-related gene. The substitutions in *RIB1* and *SOL3* were located within conserved regions of the respective proteins, and both genes showed lower transcript abundance in M70. In contrast, the ML-like protein and ABC transporter-related genes showed little change in transcript abundance.

A 1.69-kb interchromosomal duplicative insertion was also detected in M70. The insertion disrupted the coding region of a *CET1*-like gene encoding a Cet1-like mRNA triphosphatase and was associated with the formation of an aberrant chimeric transcript. The duplicated segment originated from a *REX2*-like gene encoding a Rex2-like oligoribonuclease and contained the predicted bZIP domain rather than representing a complete gene duplication. The *REX2*-like gene also showed lower transcript abundance in M70.

Together, these genomic alterations affected genes associated with cofactor biosynthesis, central carbon metabolism, transport, and RNA-related processes, providing a set of candidate genetic changes for further interpretation of the adaptive phenotype of M70.

### 3.5. Transcriptomic and Metabolomic Analyses Revealed Metabolic Alterations Associated with Long-Term Adaptation in M70

**Figure 5:**
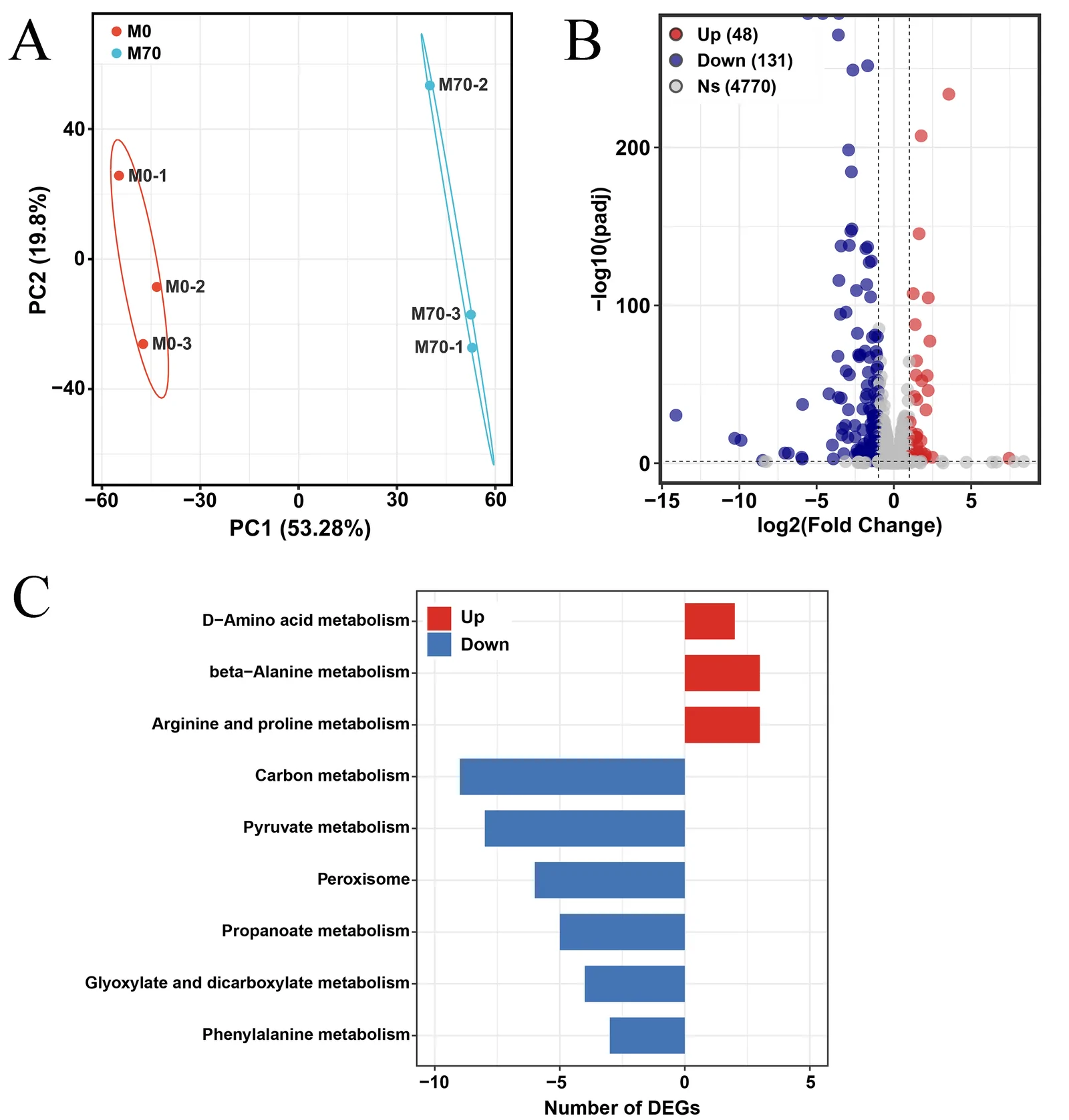
Comparative transcriptomic analysis of strains M0 and M70 during molasses cultivation. (**A**) Principal component analysis (PCA) of RNA-seq samples. (**B**) volcano plot of differentially expressed genes (DEGs) between M70 and M0. The horizontal dashed line marks the cutoff of padj = 0.05. The vertical dashed lines are at log2(Fold Change) = 1 and −1. (**C**) KEGG pathway enrichment analysis of upregulated and downregulated DEGs.

Comparative transcriptomic analysis was performed between M0 and M70 after cultivation in 100 g/L molasses medium. Approximately 40 million clean reads were obtained per sample, with Q30 values of 95.59–96.41% and mapping rates of 86.60–87.33%, indicating sufficient sequencing quality for subsequent analyses. Principal component analysis (PCA) clearly separated M0 and M70, with PC1 and PC2 explaining 53.28% and 19.80% of the total variance, respectively, while biological replicates clustered closely within each group (Figure 5A).

Differential expression analysis identified 179 differentially expressed genes (DEGs) in M70 relative to M0 based on |log_2_ fold change| > 1 and adjusted *p* < 0.05, including 48 upregulated and 131 downregulated genes (Figure 5B).

KEGG enrichment analysis showed distinct changes in metabolic pathways (Figure 5C). Upregulated genes were mainly enriched in beta-alanine metabolism, arginine and proline metabolism, and D-amino acid metabolism, whereas downregulated genes were enriched in pathways related to central carbon metabolism, including pyruvate metabolism, glyoxylate and dicarboxylate metabolism, and the TCA cycle.

**Figure 6:**
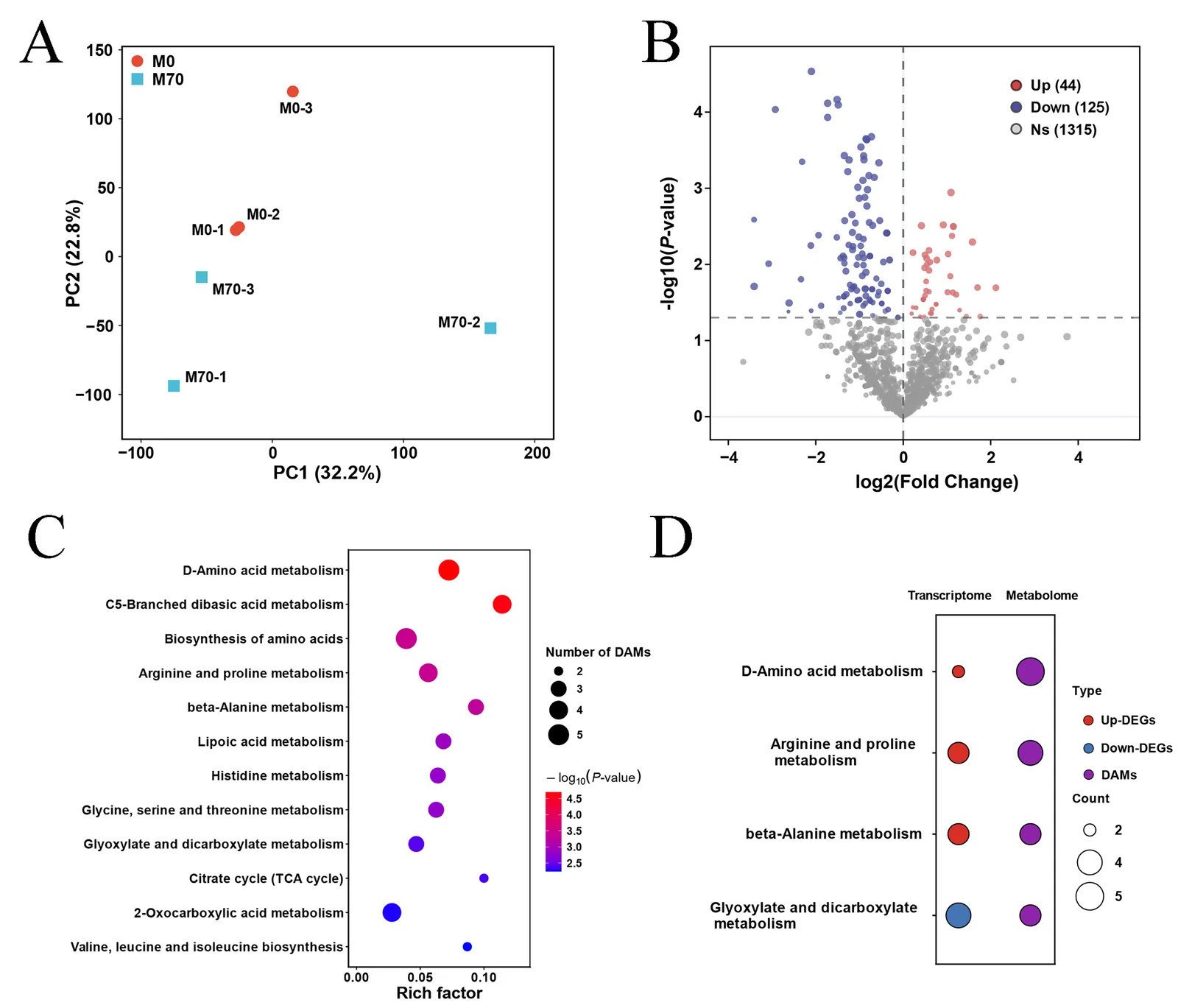
Integrated transcriptomic and metabolomic analysis of metabolic remodeling in strain M70 during adaptive evolution. (**A**) PCA of intracellular metabolomic profiles of M0 and M70. (**B**) volcano plot of differentially accumulated metabolites (DAMs). The horizontal dashed line indicates the cutoff of *p*-value = 0.05. The vertical dashed line is at log2(Fold Change) = 0. (**C**) KEGG pathway enrichment analysis of DAMs; (**D**) KEGG pathways showing concurrent changes at the transcriptomic and metabolomic levels.

Untargeted intracellular metabolomic profiling was further performed to characterize metabolic alterations in M70. A total of 1484 annotated metabolites were identified, covering amino acids, organic acids, carbohydrates, lipids, and other metabolite classes. PCA showed a clear separation between M0 and M70, with PC1 and PC2 explaining 32.2% and 22.8% of the total variance, respectively (Figure 6A). A total of 169 differentially accumulated metabolites (DAMs) were identified, including 44 increased and 125 decreased metabolites in M70 relative to M0 (Figure 6B).

KEGG enrichment analysis showed that the DAMs were mainly associated with amino acid and central carbon metabolism (Figure 6C). Significantly enriched pathways included D-amino acid metabolism, C5-branched dibasic acid metabolism, biosynthesis of amino acids, arginine and proline metabolism, glyoxylate and dicarboxylate metabolism, citrate cycle (TCA cycle), and 2-oxocarboxylic acid metabolism.

Integrated transcriptomic and metabolomic analysis identified four pathways showing concurrent changes at both the transcriptional and metabolite levels: D-amino acid metabolism, arginine and proline metabolism, beta-alanine metabolism, and glyoxylate and dicarboxylate metabolism (Figure 6D; Table S1). The first three pathways were associated predominantly with upregulated DEGs, whereas glyoxylate and dicarboxylate metabolism was associated with downregulated DEGs. Multiple DAMs were also detected in each of these pathways. Detailed gene–metabolite changes are provided in Table S1.

Collectively, the transcriptomic and metabolomic results revealed coordinated remodeling of amino acid and central carbon metabolism in M70 following long-term adaptation to high-molasses conditions.

## 4. Discussion

Many molasses-based fermentation processes employ supplementation with nitrogen sources, vitamins, or other nutrients to improve microbial growth and product formation, although direct fermentation using molasses as the sole nutrient source has also been reported in a limited number of microbial systems [19,20,21]. In the present study, strain M0 was able to grow directly in untreated molasses diluted only with water, indicating that *P. ethanolica* can obtain the nutrients required for growth without additional supplementation. However, medium composition was not systematically optimized, and whether nutrient supplementation could further improve biomass formation, sugar conversion, or fermentation performance remains to be determined.

ALE further improved the phenotypic performance of the strain. During evolution, stepwise increases in molasses concentration were accompanied by transient fluctuations in OD_560_ followed by recovery and stabilization. Such fluctuations may partly reflect population heterogeneity, as subpopulations with different fitness can shift in relative abundance under changing selection pressures [22]. M70 subsequently exhibited improved growth under high molasses concentrations and enhanced tolerance to ethanol, oxidative, salt, and temperature stresses (Figure 4). This improved growth phenotype remained stable over ten additional serial transfers, indicating short-term phenotypic stability under the tested laboratory conditions. However, long-term genetic stability, particularly at the genomic level, was not evaluated in the present study. M70 also showed lower residual sucrose and increased glycerol and ethanol accumulation during molasses cultivation (Figure 3). The transient accumulation of glycerol may be related to osmotic adaptation, consistent with its role as a compatible solute in yeast [23].

M70 also showed improved growth on glucose, fructose, and sucrose as individual carbon sources, while these major sugars were progressively consumed during molasses cultivation. Although no obvious sequential sugar-utilization pattern was observed, the present data are insufficient to conclude that carbon catabolite repression was absent or alleviated because its regulatory basis was not directly examined. Similarly, although ethanol accumulation was higher in M70, ethanol production was not the primary objective of this study and fermentation conditions were not optimized for ethanol yield or productivity. Previous studies have demonstrated the application potential of *P. ethanolica* (also reported as *Candida ethanolica*) in vinegar fermentation and bio-based product development, including aroma enhancement and production of bioactive α-mannan [24,25]. Improved direct utilization of molasses may therefore broaden the potential applications of this non-conventional yeast. However, the lack of established genetic manipulation tools for M0 and M70 currently limits direct functional validation of the adaptive changes identified here.

Comparative whole-genome resequencing identified several candidate genetic changes in M70, including coding and non-coding SNPs and an interchromosomal duplicative insertion. The occurrence of multiple candidate variants is consistent with previous ALE studies showing that complex adaptive phenotypes often arise from the combined effects of multiple genetic changes rather than a single dominant mutation [26]. Among these variants, substitutions in *RIB1* and *SOL3*, which are associated with riboflavin/cofactor biosynthesis and the pentose phosphate pathway, respectively, may be relevant to the metabolic remodeling observed in M70. A notable structural variation disrupted a *CET1*-like gene encoding an mRNA triphosphatase, while the inserted segment originated from a *REX2*-like region containing a predicted bZIP domain and was associated with an aberrant chimeric transcript. These changes may influence RNA-related regulation, but their direct contributions to the evolved phenotype remain to be experimentally validated. Accordingly, the identified variants should be regarded as candidate adaptive changes rather than causal mutations.

Transcriptomic and metabolomic analyses further indicated extensive remodeling of amino acid and central carbon metabolism. Pathways related to D-amino acid metabolism, arginine and proline metabolism, and beta-alanine metabolism showed concurrent changes at both the transcriptional and metabolite levels, suggesting altered amino acid and nitrogen-associated metabolism during long-term adaptation [27]. Such changes may also contribute to stress adaptation, particularly because proline and related metabolites are frequently involved in cellular responses to osmotic and oxidative stress. In contrast, glyoxylate and dicarboxylate metabolism was characterized predominantly by downregulated DEGs, consistent with reduced reliance on alternative carbon-assimilation pathways under the sugar-rich molasses environment. Together with enrichment of pyruvate metabolism and the TCA cycle, these results suggest that ALE reshaped the balance between central carbon metabolism and amino acid-related metabolism rather than altering a single metabolic pathway.

Broader transcriptional changes supported this interpretation (Figure S5). Genes associated with gluconeogenesis, the glyoxylate cycle, and alternative carbon metabolism, including *PCKA*, *ICL*, *MDH2*, and *ARO10*, were downregulated in M70, consistent with reduced investment in alternative carbon-utilization pathways under continuous sugar-rich cultivation. In contrast, *GPD1* was upregulated, in agreement with the increased glycerol accumulation observed during molasses fermentation and supporting a possible role for glycerol metabolism in osmotic and redox adaptation. Changes in transporter, oxidative-stress-response, and lipid-metabolism genes further suggest that adaptation involved coordinated adjustment of nutrient transport, stress defense, and membrane homeostasis.

The multi-pathway nature of these changes is broadly consistent with previous ALE studies in molasses-based systems. In *Z. mobilis*, fructose-directed evolution improved molasses utilization mainly through enhanced fructose transport and redistribution of sucrose metabolism [12], whereas ALE of *Lactobacillus delbrueckii* was associated with changes in glycolysis, cofactor supply, the pentose phosphate pathway, amino acid metabolism, and fatty acid utilization [15]. Transcriptomic analysis of molasses-adapted *Streptomyces albulus* similarly revealed changes in sucrose metabolism, stress responses, and amino acid transport [28]. In addition, studies in *S. cerevisiae* have shown that ionic imbalance and cell-envelope stress contribute to molasses inhibition and can be alleviated through remodeling of membrane-associated functions, transport systems, and stress-response pathways [13,29]. M70 therefore appears to have acquired a similarly multifaceted adaptive phenotype involving central carbon allocation, amino acid metabolism, glycerol-associated osmotic adaptation, and cellular stress responses. However, because functional genetic validation is currently unavailable, these mechanisms should be regarded as a working model derived from the integrated genomic, transcriptomic, metabolomic, and phenotypic evidence.

**Figure 7:**
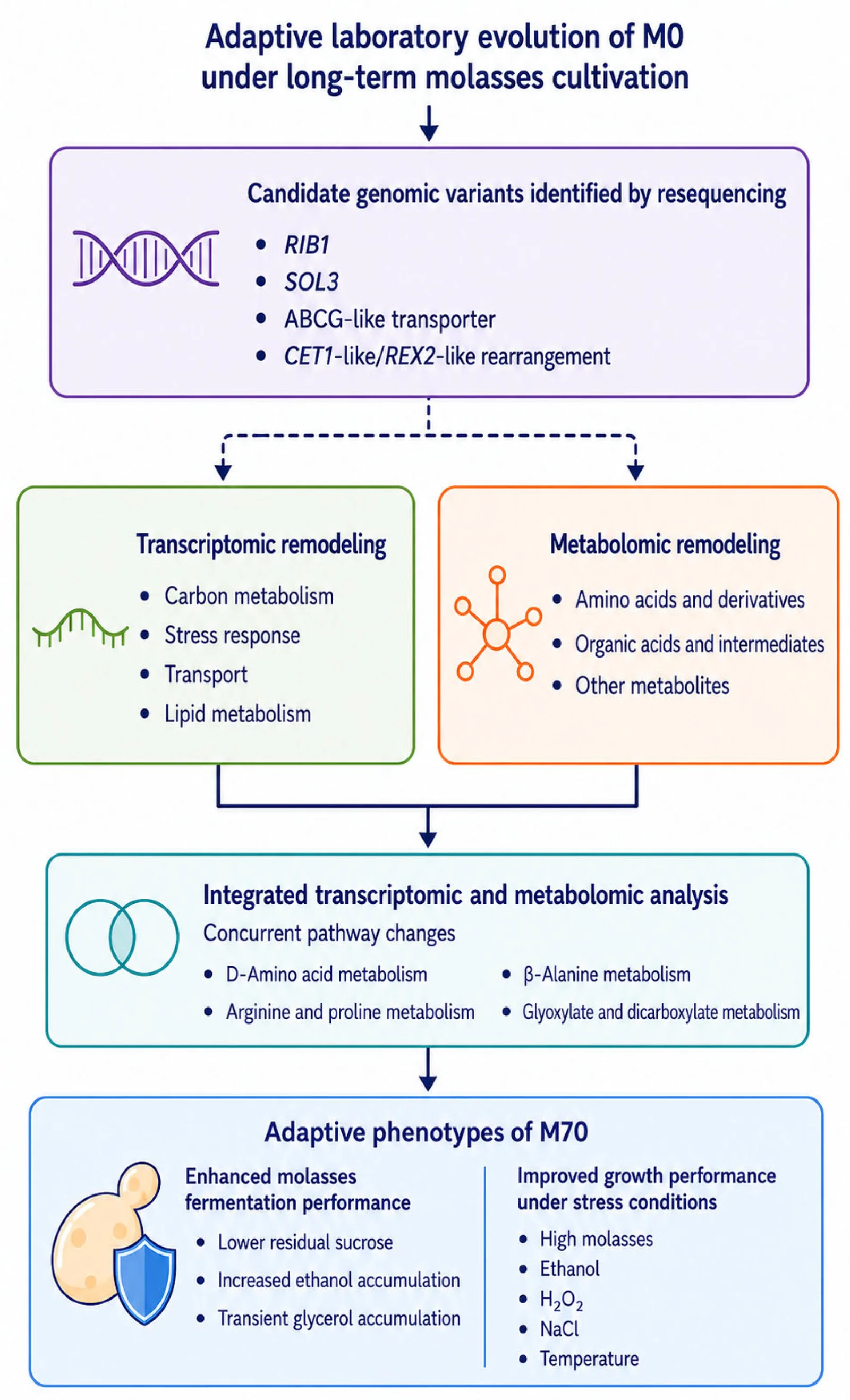
Proposed model of metabolic and physiological adaptation of strain M70 during long-term molasses cultivation. Dashed arrows indicate potential associations between candidate genomic variants identified by resequencing and downstream transcriptional and metabolic remodeling, rather than direct causal relationships.

In summary, the phenotypic, genomic, transcriptomic, and metabolomic data support a model of multilevel adaptive remodeling in M70 during long-term molasses cultivation (Figure 7). Adaptive evolution was accompanied by candidate genomic variations and coordinated transcriptional and metabolic changes involving central carbon and amino acid metabolism, nutrient transport, membrane-associated processes, and stress responses. These changes were associated with improved growth and fermentation performance in molasses and enhanced tolerance to multiple environmental stresses, suggesting increased physiological flexibility of *P. ethanolica* following ALE. Collectively, this study provides insights into the molecular basis of adaptation in a non-conventional yeast and establishes a foundation for the further development of *P. ethanolica* for molasses-based bioconversion.

## Figures and Tables

**Table 1:** Carbon source utilization profile of strain M0.

Utilization Level ^1^	Carbon Sources
+++	α-D-Glucose; Glycerol; fumaric acid; succinic acid; L-malic acid; α-ketoglutaric acid
++	Sucrose; D-galactose; D-arabitol; L-arabinose; xylitol; m-inositol plus D-xylose; 1,2-propanediol plus D-xylose
+	D-melibiose; stachyose; D-trehalose; D-arabinose; formic acid; propionic acid; D-gluconic acid; L-glutamic acid; maltose

^1^ Substrate utilization was semi-quantitatively classified based on endpoint OD_590_ values obtained using Biolog YT MicroPlates. The number of “+” symbols indicates the relative utilization level: +++, strong; ++, moderate; +, weak.

**Table 2:** Genomic variants identified in M70 and corresponding transcriptomic changes.

Variant	Gene	Functional Annotation	Variant Class/Predicted Effect	log_2_FC (M70/M0)	Adjusted *p*
bctg00000001:1543174 G > A	*FLO11*-like	Flo11-like cell surface protein	SNP; intronic variant	NA	NA
bctg00000000:2826482 G > A	*RIB1*	GTP cyclohydrolase II	SNP; D139N missense	−0.646	1.35 × 10^−10^
bctg00000003:1426087 A > G	*SOL3*	6-phosphogluconolactonase	SNP; S214G missense	−0.358	7.33 × 10^−10^
bctg00000008:644929 C > G	ML-like	ML-like/TRP channel protein	SNP; S105T missense	−0.085	0.502
bctg00000008:1511844 G > T (multiallelic G > A/T)	ABCG-like	ABC transporter-related protein	Multiallelic SNP; G364V (T allele)	−0.071	0.671
1.69-kb interchromosomal duplicative insertion: bctg00000000:2566684–2568370 → bctg00000008:974807–974811	*CET1*-like	Cet1-like mRNA triphosphatase	Interchromosomal duplicative insertion; early coding-sequence disruption	−0.139	0.365
Source region of the 1.69-kb insertion	*REX2*-like	Rex2-like oligoribonuclease with predicted bZIP domain	Partial duplication of aa 293–691, retaining the complete predicted bZIP domain	−0.970	3.73 × 10^−38^

Note: Variant positions are reported relative to the M0 reference genome assembly, with contig IDs followed by genomic coordinates. SNP, single-nucleotide polymorphism; NA, not available. Transcriptomic values represent log_2_ fold changes in M70 relative to M0. Adjusted *p* values were obtained from transcriptomic differential-expression analysis.

## Data Availability

The genome assembly of strain M0 has been deposited in the Genome Warehouse (GWH) of the National Genomics Data Center (NGDC) under accession number GWHGEDG00000000.1 within BioProject PRJCA041809. The raw whole-genome resequencing and RNA-seq data have been deposited in the Genome Sequence Archive (GSA) of the NGDC under the same BioProject, with accession numbers CRA046899 and CRA044850, respectively. Processed metabolomic data relevant to this study are provided in the Supplementary Materials. The raw metabolomic data have not been deposited in a public repository and are available from the corresponding author upon reasonable request.

## Supplementary Materials

The following supporting information can be downloaded at https://www.mdpi.com/article/10.3390/microorganisms14092037/s1. Figure S1: Screening of isolates based on growth performance in molasses medium; Figure S2: Screening of evolved isolates and growth characterization of strain M70; Figure S3: Stability evaluation of the evolved strain M70 during serial transfers in molasses medium; Figure S4: Genome heterozygosity assessment of the parental strain M0; Figure S5: Heatmap of representative differentially expressed genes associated with molasses adaptation in M70; Table S1: Differentially expressed genes and metabolites mapped to the selected KEGG pathways; Table S2: Differential metabolites identified between M0 and M70 based on LC-MSMS analysis.

## Abbreviations

The following abbreviations are used in this manuscript:

ALE	Adaptive laboratory evolution
ANI	Average nucleotide identity
DAMs	Differentially accumulated metabolites
DEGs	Differentially expressed genes
PCA	Principal component analysis
SNP	Single-nucleotide polymorphism
VIP	Variable importance in projection
