# Supplementary material for: Adaptive Laboratory Evolution of the Non-Conventional Yeast Pichia ethanolica for Improved Molasses Utilization Reveals Multilevel Adaptive Remodeling
Source: Microorganisms. 2026 Sep 12;14(9):2037. doi: 10.3390/microorganisms14092037 (PMC13609567; doi:10.3390/microorganisms14092037)
Supplement: Supplementary file 1 [file microorganisms-14-02037-s001.zip › Supplementary Material.pdf]

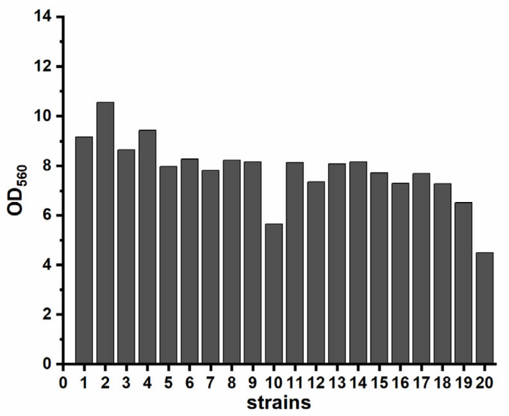

Figure S1. Screening of isolates based on growth performance in molasses medium. Twenty isolates selected from molasses agar plates were cultivated in 5 mL of 4% (w/v) molasses medium at 30 °C and 150 rpm for 48 h. Growth was evaluated by measuring the final OD_{560}. Numbers on the x-axis indicate individual isolates. Isolate No. 2 exhibited the highest OD_{560} and was designated as strain M0 for subsequent studies.

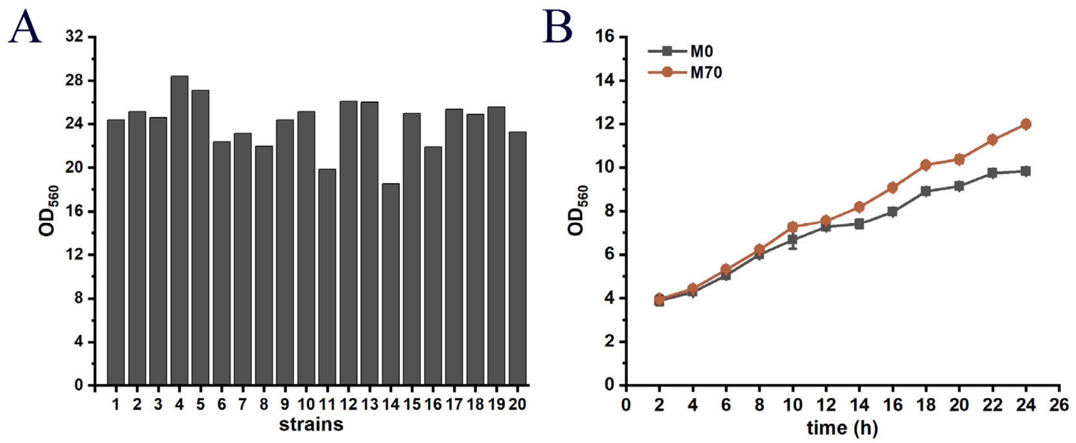

Figure S2. Screening of evolved isolates and growth characterization of strain M70. (**A**) Growth performance of 20 isolates obtained from the final evolved population after 48 h of cultivation in liquid molasses medium; (**B**) Growth curves of the parental strain M0 and evolved strain M70 in YPD medium. Data are presented as mean ± standard deviation.

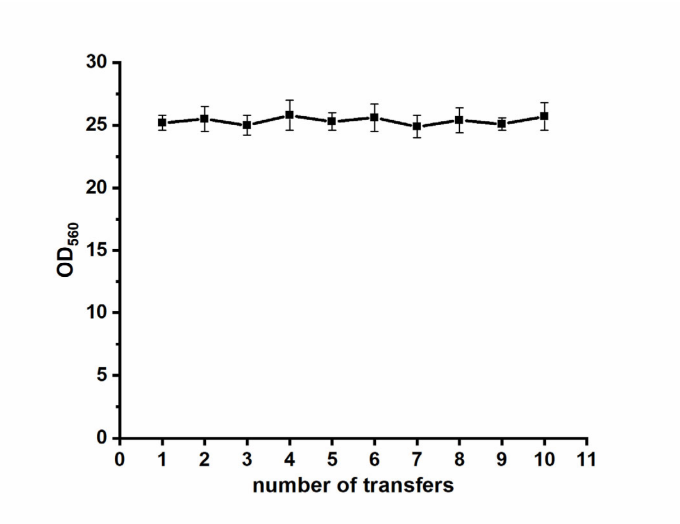

Figure S3. Stability evaluation of the evolved strain M70 during serial transfers in molasses medium. Strain M70 was serially transferred every 72 h for 10 consecutive transfers in 100 g/L molasses medium under the same cultivation conditions used previously (30 °C, 150 rpm). Growth performance at each transfer was evaluated by measuring the final OD_{560} value at the end of cultivation. No obvious decline in growth performance was observed over the 10 transfers. Data are presented as mean ± standard deviation.

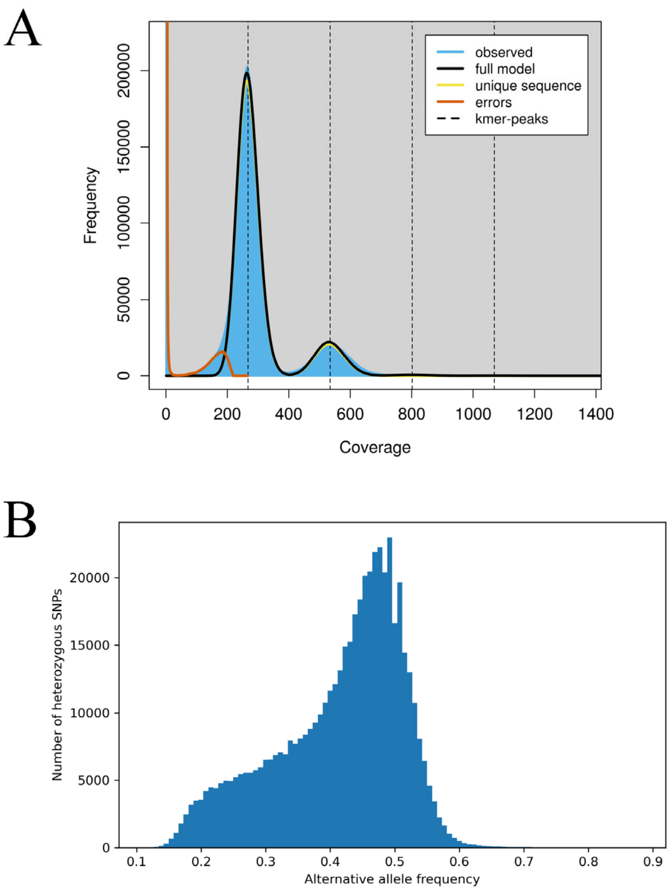

Figure S4. Genome heterozygosity assessment of the parental strain M0. (**A**) GenomeScope analysis of the 21-mer frequency distribution; (**B**) Allele-frequency distribution of heterozygous SNPs. GenomeScope analysis revealed a bimodal k-mer distribution and an estimated heterozygosity of 6.67% under a diploid model. Consistently, 518,111 filtered heterozygous SNPs showed a predominant allele-frequency peak around 0.5, further supporting the highly heterozygous genomic background of M0.

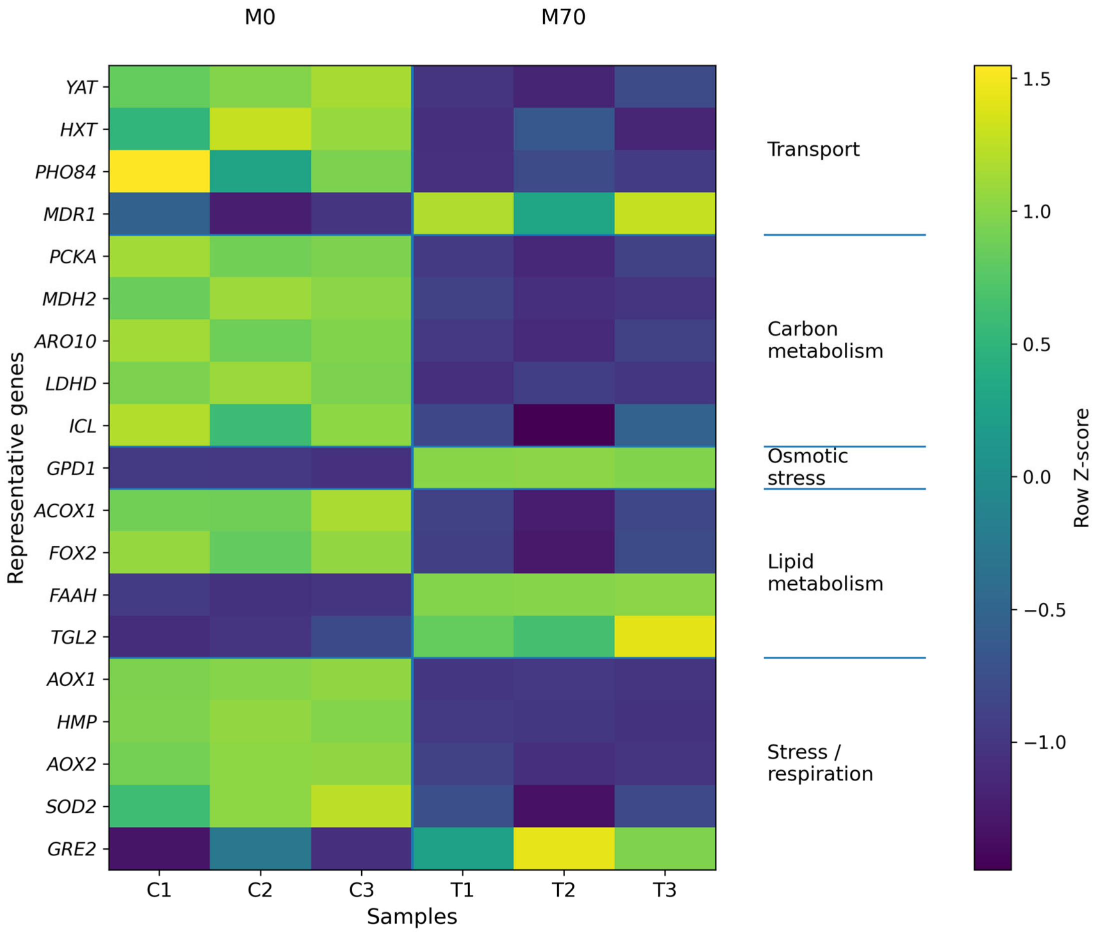

Figure S5. Heatmap of representative differentially expressed genes associated with molasses adaptation in M70. The heatmap displays the expression patterns of selected DEGs between the parental strain M0 and the evolved strain M70. Genes were selected based on their functional relevance to carbon metabolism, transport, osmotic stress response, lipid metabolism, and stress/respiration-related processes. Expression values were normalized by row-wise Z-score transformation. Color gradients indicate relative expression levels, with yellow and purple representing higher and lower expression levels, respectively.

Table S1. Differentially expressed genes and metabolites mapped to the selected KEGG pathways.

Pathway	Omics	Feature name	KEGG KO / Compound ID	Annotation	Regulation	log_2FC (M70/M0)
D-Amino acid metabolism	Transcriptome	DAO	K00273	D-amino acid oxidase	Up-regulated	1.803
		GBH	K25365	Guanidinobutyrase / D-arginase	Up-regulated	1.049
	Metabolome	Proline	C00148	DAM	Increased	0.459
		Alanylalanine (Ala-Ala)	C00993	DAM	Decreased	-0.912
		Glycine	C00037	DAM	Increased	1.092
		D-Glutamine	C00819	DAM	Increased	1.115
		2-Oxoglutarate	C00026	DAM	Decreased	-0.854
Arginine and proline metabolism	Transcriptome	DAO	K00273	D-amino acid oxidase	Up-regulated	1.803
		GBH	K25365	Guanidinobutyrase / D-arginase	Up-regulated	1.049
		FMS1	K13367	Polyamine oxidase	Up-regulated	1.170
	Metabolome	Proline	C00148	DAM	Increased	0.459
		Homocarnosine	C00884	DAM	Decreased	-1.519
		4-Guanidinobutyric acid	C01035	DAM	Decreased	-1.096

Pathway	Omics	Feature name	KEGG KO / Compound ID	Annotation	Regulation	log_2FC (M70/M0)
beta-Alanine metabolism	Transcriptome	N-Acetylputrescine	C02714	DAM	Decreased	-0.700
		PuuE	K00823	4-Aminobutyrate aminotransferase	Up-regulated	1.764
		AOC	K00276	Primary amine oxidase	Up-regulated	1.378
		FMS1	K13367	Polyamine oxidase	Up-regulated	1.170
	Metabolome	Uracil	C00106	DAM	Decreased	-0.998
		Carnosine	C00386	DAM	Decreased	-1.018
		Anserine	C01262	DAM	Decreased	-1.352
Glyoxylate and dicarboxylate metabolism	Transcriptome	MDH2	K00026	Malate dehydrogenase	Down-regulated	-1.456
		MDH2	K00026	Malate dehydrogenase	Down-regulated	-1.430
		ICL	K01637	Isocitrate lyase / methylisocitrate lyase	Down-regulated	-1.146
		ACS	K01895	Acetyl-CoA synthetase	Down-regulated	-1.081
	Metabolome	cis-Aconitic acid	C00417	DAM	Decreased	-1.039
		Glycine	C00037	DAM	Increased	1.092
		2-Oxoglutarate	C00026	DAM	Decreased	-0.854

Note: DEGs, differentially expressed genes; DAMs, differentially accumulated metabolites. Features were mapped to KEGG pathways according to functional annotation. Positive and negative log2FC values indicate increased and decreased abundance/expression, respectively. Metabolites assigned to multiple KEGG pathways are shown repeatedly.
